# Validity of Bioelectrical Impedance Analysis for the Assessment of Body Composition in Patients With Systemic Sclerosis

**DOI:** 10.1002/jcsm.70273

**Published:** 2026-03-26

**Authors:** Lucas Denardi Dória, Rafaela Cavalheiro do Espírito Santo, Vanessa Hax, André Luiz Mallmann, Leonardo Peterson dos Santos, Stephanie Pilotti, Daniel Nóbrega de Moraes, Bruno Eduardo Lara Da Silva, Vinícius Hammel Lovison, Ronaldo Legati Junior, Poliana Espíndola Correia, Tayane Muniz Fighera, Fernando Gerchman, Poli Mara Spritzer, Ricardo Machado Xavier, Rafael Mendonça da Silva Chakr

**Affiliations:** ^1^ Division of Rheumatology Hospital de Clínicas de Porto Alegre (HCPA) Porto Alegre Rio Grande do Sul Brazil; ^2^ Postgraduate Program in Medicine: Medical Science Universidade Federal do Rio Grande do Sul (UFRGS) Porto Alegre Brazil; ^3^ Faculty of Health Sciences Klaipeda University Klaipėda Lithuania; ^4^ Division of Endocrinology Hospital de Clínicas de Porto Alegre (HCPA) Porto Alegre Rio Grande do Sul Brazil

**Keywords:** agreement, bioelectrical impedance analysis, bioimpedance (BIA), body composition, cut‐off point, fat mass, fat‐free mass, muscle mass, myopenia, systemic sclerosis (SSc)

## Abstract

**Background:**

Systemic sclerosis (SSc) is an autoimmune disease characterized by vasculopathy and progressive fibrosis. Reduced muscle mass is common and contributes to myopenia, sarcopenia and adverse clinical outcomes. Dual‐energy X‐ray absorptiometry (DXA) is the most widely used method in clinical research and is recommended by international guidelines as the reference standard for muscle mass evaluation in sarcopenia. However, its accessibility in routine clinical practice is limited. Bioelectrical impedance analysis (BIA) is a validated method for assessing body composition due to its affordability, ease of use and non‐invasive nature. Nevertheless, variability across devices and assessment protocols may influence measurement accuracy, underscoring the need for validation in specific clinical populations. This study compared body composition measured by BIA and DXA and evaluated the diagnostic accuracy of BIA for detecting myopenia in SSc.

**Methods:**

In this cross‐sectional study, patients with SSc underwent body composition assessment by BIA (InBody 370 s) and DXA (Lunar Prodigy Primo). Fat mass (FM), fat mass index (FMI), fat‐free mass (FFM), fat‐free mass index (FFMI), appendicular skeletal muscle mass (ASM), appendicular skeletal muscle mass index (ASMI) and ASM adjusted by body mass index (ASM/BMI) were assessed. Myopenia was defined according to established muscle mass criteria. Agreement was evaluated using Bland–Altman analysis and Lin's concordance correlation coefficient (CCC). Diagnostic performance was assessed using receiver operating characteristic (ROC) curves.

**Results:**

One hundred patients with SSc were included (91% women; mean age 60.1 ± 11.5 years; median disease duration 11.5 [5.0–20.3] years). Disease subtypes comprised 23% diffuse cutaneous, 61% limited cutaneous and 16% sine scleroderma. BIA demonstrated excellent agreement with DXA (CCC > 0.900) for ASM/BMI [Δ = 0.032; 95% CI 0.019–0.045], FM (Δ = −0.401; 95% CI −0.804 to 0.003), FMI (Δ = −0.190; 95% CI −0.366 to −0.015) and FFM (Δ = −0.010; 95% CI −0.415 to 0.395). Good concordance (CCC 0.750–0.900) was observed for ASM (Δ = 0.803; 95% CI 0.518–1.088). In women, ROC analysis showed high discriminative ability, with AUCs of 0.956 (95% CI 0.908–1.000) for ASM, 0.878 (95% CI 0.763–0.993) for ASMI and 0.969 (95% CI 0.935–1.000) for ASM/BMI. The optimal ASM/BMI cut‐off (0.506) yielded 100% sensitivity and 90.1% specificity (PPV 55.6%, NPV 100%).

**Conclusion:**

BIA showed high agreement with DXA and strong diagnostic accuracy for detecting myopenia in SSc, particularly in women. These findings support BIA as an accessible tool for body composition assessment in SSc when standardized conditions and validated sex‐specific cut‐offs are applied.

## 1 Introduction

Systemic sclerosis (SSc) is an autoimmune disease characterized by a triad of autoimmune activation, vasculopathy, and cutaneous or visceral fibrosis [1, 2]. Clinical factors such as chronic inflammation, immobilization due to joint involvement and contractures, renal failure, early menopause and the use of glucocorticoid medications are associated with body composition changes in these patients [3, 4, 5, 6]. Previous studies demonstrated that SSc patients exhibit a decrease in fat mass, fat‐free mass (FFM), muscle mass and bone mineral density when compared with healthy controls [7, 8].

Changes in body composition, such as an increase in fat mass, have previously been shown to be inversely associated with physical activity levels and inflammation in SSc [9, 10]. Furthermore, a reduction in FFM and loss of lean mass may lead to conditions such as malnutrition and sarcopenia [11, 12]. The European Society of Clinical Nutrition and Metabolism (ESPEN) defines malnutrition based on low body mass index (BMI), weight loss or low FFM [13]. In patients with SSc, the prevalence of malnutrition using the ESPEN criteria ranges from 8% to 18% and is consistently associated with gastrointestinal and pulmonary involvement [14, 15, 16]. A decrease in appendicular skeletal muscle (sum of lean mass from the upper and lower limbs) may lead to sarcopenia [11, 12]. In SSc, the prevalence of sarcopenia is 22% (95% CI: 17%, 28%) and it has been associated with functional capacity, vascular changes, skin scores and increased inflammatory markers [7, 10, 12].

An accurate assessment of malnutrition and sarcopenia requires precise body composition analysis [7, 13, 17]. Diagnosis of these conditions may potentially lead to the implementation of specific therapeutic interventions, including pharmacological and non‐pharmacological treatments. Although methods such as computed tomography, magnetic resonance imaging and dual‐energy X‐ray absorptiometry (DXA) offer high validity [17]. However, despite their high validity—that is, the accuracy with which they reflect true values—, these methods are often inaccessible, expensive and require specialized technical skills [17]. Bioelectrical impedance analysis (BIA) has been identified as a simpler, non‐invasive alternative. BIA employs low‐frequency electrical impedance to estimate body fluid volume and is considered an accessible method with minimal technical requirements [17, 18]. However, its application in clinical populations has limitations. Variations across device models, proprietary predictive equations and measurement protocols can compromise measurement accuracy, highlighting the need for disease‐specific validation, particularly in SSc. In accordance with current consensus, the Global Leadership Initiative in Sarcopenia (GLIS) [19] recognizes BIA as an acceptable modality when reference methods are unavailable, contingent upon population‐specific validation and the implementation of standardized preassessment protocols.

Although previous research has compared BIA and DXA in patients with SSc, these investigations were largely restricted to fat mass (FM) and FFM estimations [14] or nutritional phenotyping via bioelectrical impedance vector analysis and phase angle (Di Battista et al., 2021) [20]. To our knowledge, only Spanjer et al. have evaluated BIA relative to whole‐body DXA for the assessment of FM and FFM in patients with SSc [14]. In their study, the authors evaluated reliability—the consistency of results across repeated measures—using the intraclass correlation coefficient (ICC) to examine agreement between BIA and DXA via the manufacturer's equation. They reported ICC values of 0.908 (95% CI: 0.712, 0.959) and FFM 0.956 (95% CI: 0.921, 0.974) for FFM. Since FFM encompasses the non‐fat component of the body composition, comprising both lean mass tissue and bone mass, it is relevant to study skeletal muscle mass specifically as it is a critical criterion for diagnosing sarcopenia. Consequently, the present study expands this line of research by evaluating a broader range of body composition parameters—fat‐ and muscle‐related indices—and by determining the validity, agreement and diagnostic accuracy of BIA relative to DXA for the detection of myopenia in patients with SSc.

The primary objective of this study was to compare body composition measurements (fat mass, FFM, muscle mass and their indices) obtained by BIA and DXA in patients with SSc and to assess the overall diagnostic performance of BIA for the identification of myopenia.

## 2 Methods

### 2.1 Study Design and Participants

This was a cross‐sectional study of 100 patients with SSc conducted in accordance with the 2015 STARD Reporting Guidelines for Studies of Diagnostic Accuracy [21]. The study was conducted at the Hospital de Clínicas de Porto Alegre (HCPA), a tertiary public hospital in southern Brazil. The patients had a mean age of 60.1 ± 11.5 years, of which 91% were female. The study was approved by the HCPA Institutional Review Board (IRB) (registration number 2021‐0504) and adhered to the ethical principles of the Declaration of Helsinki. From February 2022 to June 2023, participants with SSc were consecutively enrolled during their clinical visits and were invited on a convenience basis. All participants provided written informed consent prior to enrollment.

The study included patients with SSc from the HCPA who met the 2013 American College of Rheumatology and European League Against Rheumatism (ACR/EULAR) criteria for SSc or the 2001 criteria for early SSc proposed by LeRoy and Medsger [22]. The presence of metal prostheses was not regarded as an exclusion factor. Exclusion criteria encompassed conditions such as overlapping autoimmune diseases, the use of a pacemaker, digital contractures (claw hands) or any other limitation precluding BIA assessment. These criteria are consistent with those established by our study group [11].

### 2.2 Clinical Assessment

Sociodemographic and clinical characteristics were systematically extracted from medical records, including: colour or ethnicity as self‐reported by the Brazilian Institute of Geography and Statistics (*Instituto Brasileiro de Geografia e Estatística*—IBGE), age (years old), disease duration (defined by the time since the first non‐Raynaud's symptom), disease subtype (diffuse cutaneous SSc—involving the skin of the trunk and extremities; limited cutaneous SSc—restricted to the extremities and/or face; or sine scleroderma), smoking status and treatment regimen (polypharmacy: 5 or more medications per day; or current glucocorticoid use). During routine hospital visits, laboratory parameters including the measurement of C‐reactive protein (CRP), vitamin D (25‐hydroxyvitamin D), antinuclear antibodies (ANA), anti‐tpoisomerase I antibodies (Scl‐70) and anti‐centromere antibodies (ACA) were measured. Results obtained within 1 month prior to the study visit were utilized, employing validated methods such as immunoturbidimetry, chemiluminescent immunoassays and immunofluorescence. Pulmonary function tests and echocardiography data were gathered from the year preceding the visit. On the day of the study visit, skin disease severity was assessed using the modified Rodnan skin score (mRSS) [23] and the European Scleroderma Trials & Research Group (EUSTAR) disease index [24]. Functional capacity was assessed using the Health Assessment Questionnaire‐Disability Index (HAQ‐DI) [25, 28].

### 2.3 Assessment of Body Composition

Body composition was evaluated using DXA and BIA. To ensure measurement standardization, patients were contacted 1 week before the assessments and provided with specific instructions. Both assessments were performed on the same day. Standardized protocols included fasting for at least 4 h, avoiding fluids for 2 h before the measurement, urinating before the assessment, avoiding physical exercise for at least 48 h, not being in the middle of menstruation and not wearing heavy clothing or metal accessories. Before each assessment, patients were asked to confirm adherence to the pre‐established pre‐evaluation instructions under the supervision of a trained and experienced evaluator. No participants reported noncompliance with the fasting requirements. Regarding hydration, some patients reported mild symptoms such as fatigue and dry mouth; therefore, they were allowed to moisten their lips to minimize discomfort. All patients were instructed to urinate immediately before the evaluations. No clinically relevant oedema was detected on inspection at the time of assessment.

DXA measurements were performed using the Lunar Prodigy Primo (GE Healthcare, Fan Beam 4500A) with Encore version 14.10 software (Radiation Corporation, Madison, WI, USA). Calibration was conducted using a manufacturer‐supplied phantom. Scans were performed by specialized technicians with the patient in the supine position, arms at the sides and legs slightly apart. The quality‐control protocol showed a coefficient of variation < 2%, indicating excellent measurement reliability [26, 27].

BIA was performed using the InBody 370S (Biospace Co. Ltd., Seoul, South Korea), a multifrequency analyser with tetrapolar tactile electrodes. The device executes 15 impedance measurements at three frequencies (5, 50 and 250 kHz) over five body segments: right arm, left arm, trunk, right leg and left leg. Participants stood barefoot on the platform maintaining standardized hand‐electrode contact while avoiding movement or speech. Body composition parameters (including fat mass, FFM and lean mass) were estimated using multifrequency manufacturer‐derived proprietary equations, as raw impedance vector data were not available for analysis. Appendicular skeletal muscle mass (ASM) was manually calculated as the sum of lean mass from the upper and lower limbs, since this variable is not directly provided by the manufacturer. We acknowledge that the use of proprietary algorithms may limit comparability with other BIA models. All measurements were supervised or performed by a trained examiner to ensure accuracy.

Both modalities provided quantitative measures of FM, FFM and muscle mass. Derived indices included the fat mass index (FMI = FM/height^2^) and fat‐free mass index (FFMI = FFM/height^2^). ASM was obtained by summing the lean mass of the arms and legs. Adjusted muscle mass was expressed as appendicular skeletal muscle mass index (ASMI = ASM/height^2^) and ASM divided by BMI (ASM/BMI). The cut‐off points used to define low muscle mass were derived from established international guidelines. For ASM and ASMI, thresholds were based on the European Working Group on Sarcopenia in Older People 2 (EWGSOP2) recommendations, whereas ASM/BMI cut‐offs followed the criteria proposed by the Foundation for the National Institutes of Health (FNIH). Accordingly, myopenia was considered as low muscle mass [28], which was defined as ASM < 20 kg, ASMI < 7.0 kg/m^2^ and ASM/BMI < 0.789 for men, and ASM < 15 kg, ASMI < 5.5 kg/m^2^ and ASM/BMI < 0.512 for women [29]. Obesity was defined by a BMI of more than 30 kg/m^2^ and malnutrition was defined in accordance with the European Society of Clinical Nutrition and Metabolism [13]: either a BMI < 18.5 kg/m^2^ or unintentional weight loss (greater than 10% of usual body weight or greater than 5% over 3 months) combined with either a BMI < 20 kg/m^2^ for individuals under 70 years old or < 22 kg/m^2^ for those aged 70 and older or a fat‐free mass index (FFMI) < 15 kg/m^2^ for women and < 17 kg/m^2^ for men.

### 2.4 Statistical Analysis

The sample size was determined based on Bland and Altman's [30] for assessing agreement between two clinical measurement methods. These authors suggest a sample size of 100, which provides a standard error for the 95% limits of agreement, resulting in a confidence interval (CI) of approximately ±0.34 *s*, where *s* represents the standard deviation. The Shapiro–Wilk method was used to test for normality. Results are presented as mean ± standard deviation (SD), median (interquartile range, IQR) or number (%), as appropriate. Agreement between DXA and BIA body composition measurements was assessed using the Concordance Correlation Coefficient (CCC) by Lin [31] and Bland–Altman plot, which involves constructing an agreement plot of mean (DXA + BIA/2) versus difference (DXA—BIA) and calculating the limit of agreement. Based on Spanjer et al., a maximum systematic bias of 10% was defined as the threshold for clinically acceptable agreement between methods in patients with SSc [14]. The sensitivity, specificity, positive predictive value, negative predictive value and overall accuracy of muscle mass by BIA were evaluated using ROC curves and the Youden index. The area under the ROC curve (AUC) and 95% CI were calculated for all components of muscle mass by BIA. CCC was interpreted as poor concordance if CCC < 0.500, moderate concordance if between 0.500 and 0.7500, good concordance if between 0.750 and 0.900 and excellent concordance if > 0.900, significance *p* < 0.05. Internal validation was performed to assess potential optimism in model performance, using bootstrap resampling with 1000 iterations (R package boot, version 1.3–30). Calibration was evaluated by estimating the calibration intercept and slope, with 95% bias‐corrected and accelerated (BCa) confidence intervals. Adequate calibration was defined by intercepts including 0 and slopes including 1 within the 95% CI. The Statistical Package for the Social Sciences version 18 software and the R language were used for statistical analysis and graph generation, respectively.

## 3 Results

### 3.1 Demographic and Clinical Features

**FIGURE 1:**
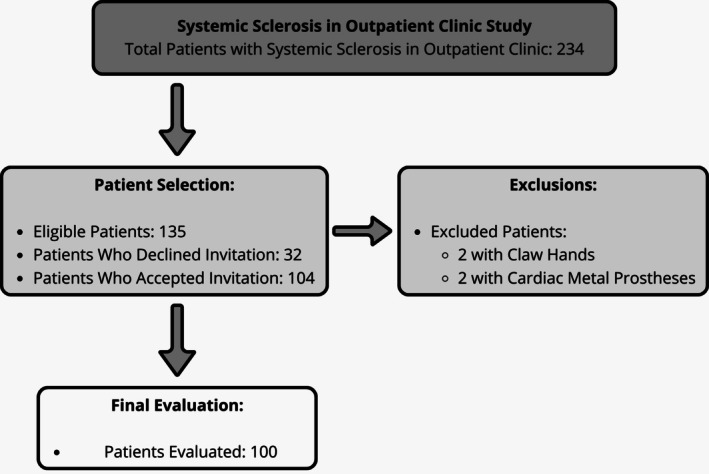
Flowchart of systemic sclerosis in outpatient clinic.

Of the 135 eligible patients identified in our outpatient clinic, 104 (77%) accepted the invitation to participate (Figure 1). No participant reported an adverse event during the BIA body composition assessment. Four participants were subsequently excluded: two due to metallic cardiac prostheses and two due to digital contractures (claw hands). Consequently, the final analysis included 100 patients with SSc. The mean age was 60.1 ± 11.5 years old, the median disease duration was 11.5 (5.0–20.3) years and there was a predominance of female patients (91%). Regarding ethnicity (IBGE self‐report), 83% identified as White, 9% as Black and 8% as mixed race. Diffuse cutaneous involvement was observed in 23%, whereas 61% had the limited subtype and 16% had *sine scleroderma*. The prevalence of autoantibodies was 94/100 (94%) for ANA, 8/87 (9%) for Anti‐Scl70 and 40/95 (40%) for anti‐centromere antibodies. The median mRSS was 4.0 (2.0–9.0) and the EUSTAR index was 1.8 (1.0–3.3). Obesity was detected in 15 (15%) patients and malnutrition in 6 (6%) patients. Metal prosthesis use was recorded in 4 (4%) of cases. Further details are shown in Table 1.

**TABLE 1:** Clinical features of patients with SSc.

Characteristics	*n* varies^a^
Age (years old), *n* = 100	60.1 ± 11.5
Female, *n* = 100	91 (91%)
Disease duration (in years), *n* = 98	11.5 (5.0–20.3)
Diffuse skin involvement, *n* = 100	23 (23%)
CRP (mg/L), *n* = 82	3.2 (1.4–7.2)
Vitamin D (ng/mL), *n* = 80	29.9 (24.5–36.5)
Polypharmacy, *n* = 91	66 (72%)
Smoking status, *n* = 100	10 (10%)
Current use of glucocorticoid, *n* = 100	8 (8%)
Hospitalized past year, *n* = 100	20 (20%)
Digital ulcers, *n* = 100	15 (15%)
BMI (kg/m^2^), *n* = 100	26.0 ± 4.8
^b^Obesity, *n* = 100	15 (15%)
^c^Malnutrition, *n* = 100	6 (6%)
FVC (% predicted), *n* = 96	82.2 ± 23.6
Reduced FVC (< 80% of predicted), *n* = 96	40 (42%)
DLCO (% predicted), *n* = 88	58.0 ± 20.0
Reduced DLCO (< 80% of predicted), *n* = 88	70 (80%)
sPAP (mmHg), *n* = 67	26.0 (23.0–30.0)
^d^Pulmonary hypertension (sPAP > 35), *n* = 67	5 (7%)
mRRS, *n* = 100	4.0 (2.0–9.0)
EUSTAR index, *n* = 79	1.8 (1.0–3.3)
Systemic arterial hypertension, *n* = 91	25 (27%)
Diabetes mellitus, *n* = 91	7 (8%)
Utilization of metal bone prosthesis, *n* = 100	4 (4%)
HAQ, *n* = 100	0.8 (0.4–1.4)

*Note:* The number of patients with available data for each variable is specified in the table. Values are expressed as mean ± standard deviation, frequency (number and percentage) or median [interquartile range (25–75)].

Abbreviations: BMI (kg/m^2^): body mass index in kilograms per square metre; CRP mg/L: C‐reactive protein in milligrams per litre; DLCO (% predicted): Diffusing Capacity of the Lungs for Carbon Monoxide as a percentage of predicted; EUSTAR: European Scleroderma Trials and Research; FVC (% predicted): forced vital capacity as a percentage of predicted; HAQ: Health Assessment Questionnaire; mRSS: Modified Rodnan Skin Score; *n*: number; sPAP: systolic pulmonary artery pressure; Vitamin D ng/mL: Vitamin D in nanograms per millilitre.

^a^ Total study population was 100 patients.

^b^ Obesity was characterized by a BMI of more than 30 kg/m^2^.

^c^ Malnutrition in accordance with European Society for Clinical Nutrition and Metabolism.

^d^ Screening for pulmonary hypertension using echocardiography.

### 3.2 Agreement Between BIA and DXA

BIA and DXA demonstrated excellent concordance (CCC > 0.900) for the following parameters: ASM/BMI [Δ = 0.032 (95% CI: 0.019, 0.045)], FM [Δ = −0.401 kg (95% CI: −0.804, 0.003)], FMI [Δ = −0.190 kg/m^2^ (95% CI: −0.366, −0.015)] and FFM [Δ = −0.010 kg (95% CI: −0.415, 0.395)]. Furthermore, ASM and FFMI showed good concordance (CCC between 0.750 and 0.900, Table 2). All mean systematic biases (Δ) were below 10% of DXA‐derived values, indicating clinically acceptable agreement.

**TABLE 2:** Comparison between DXA and BIA measurements.

	DXA	BIA	Δ (95% CI of Δ)	Δ% of DXA mean	CCC (95% CI of CCC)	*p*
ASM, kg	16.755 ± 3.359	15.952 ± 3.475	0.803 (0.518, 0.188)	4.8%	0.887^a^ (0.839, 0.922)	< 0.001
ASMI, kg/m^2^	6.768 ± 0.904	6.405 ± 0.917	0.363 (0.239, 0.487)	5.4%	0.709 (0.604, 0.789)	< 0.001
ASM/BMI	0.623^b^ (0.546–0.709)	0.591^b^ (0.531–0.687)	0.032 (0.019, 0.045)	5.1%	0.905^c^ (0.864, 0.935)	< 0.001
FM, kg	24.187 ± 9.126	24.588 ± 9.012	−0.401 (−0.804, 0.003)	1.7%	0.974^c^ (0.962, 0.982)	< 0.001
FMI, kg/m^2^	9.901 ± 3.879	10.092 ± 3.915	−0.190 (−0.366, −0.015)	1.9%	0.973^c^ (0.960, 0.982)	< 0.001
FFM, kg	39.356 ± 6.411	39.366 ± 6.880	−0.010 (−0.415, 0.395)	0.03%	0.953^c^ (0.932, 0.968)	< 0.001
FFMI. kg/m^2^	15.938 ± 1.716	15.878 ± 1.725	0.060 (−0.105, 0.224)	0.4%	0.884^a^ (0.832, 0.920)	< 0.001

*Note:* Δ: mean systematic difference (Bland–Altman bias) between DXA and BIA measurements, expressed in kilograms (kg) or kilograms per square metre (kg/m^2^). Values are expressed as mean ± standard deviation, 95% CI (confidence interval) or median [interquartile range 25–75]. All mean systematic biases (Δ) were below 10% of DXA‐derived values, indicating clinically acceptable agreement between methods.

Abbreviations: ASM, appendicular skeletal muscle mass; ASM/BMI, appendicular skeletal muscle mass divided by body mass index; ASMI, appendicular skeletal muscle index; BIA, bioelectrical impedance analysis; CCC, concordance correlation coefficient; DXA, dual‐energy X‐ray absorptiometry; FFM, fat‐free mass; FFMI, fat‐free mass index; FM, fat mass; FMI, fat mass index; *p*, *p*‐value of CCC.

^a^ CCC > 0.750–0.900 = good.

^b^ Logarithm‐based normal distribution.

^c^ CCC > 0.900 = excellent.

**FIGURE 2:**
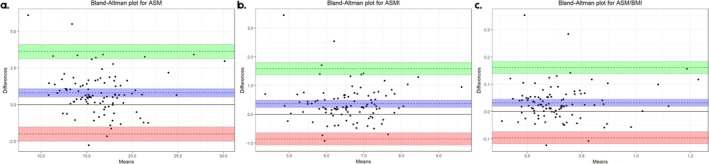
Bland–Altman plot of difference and means of muscle mass measured by DXA and BIA: (a) appendicular skeletal muscle mass (ASM), (b) appendicular skeletal muscle mass index (ASM) and (c) ASM/body mass index.

**FIGURE 3:**
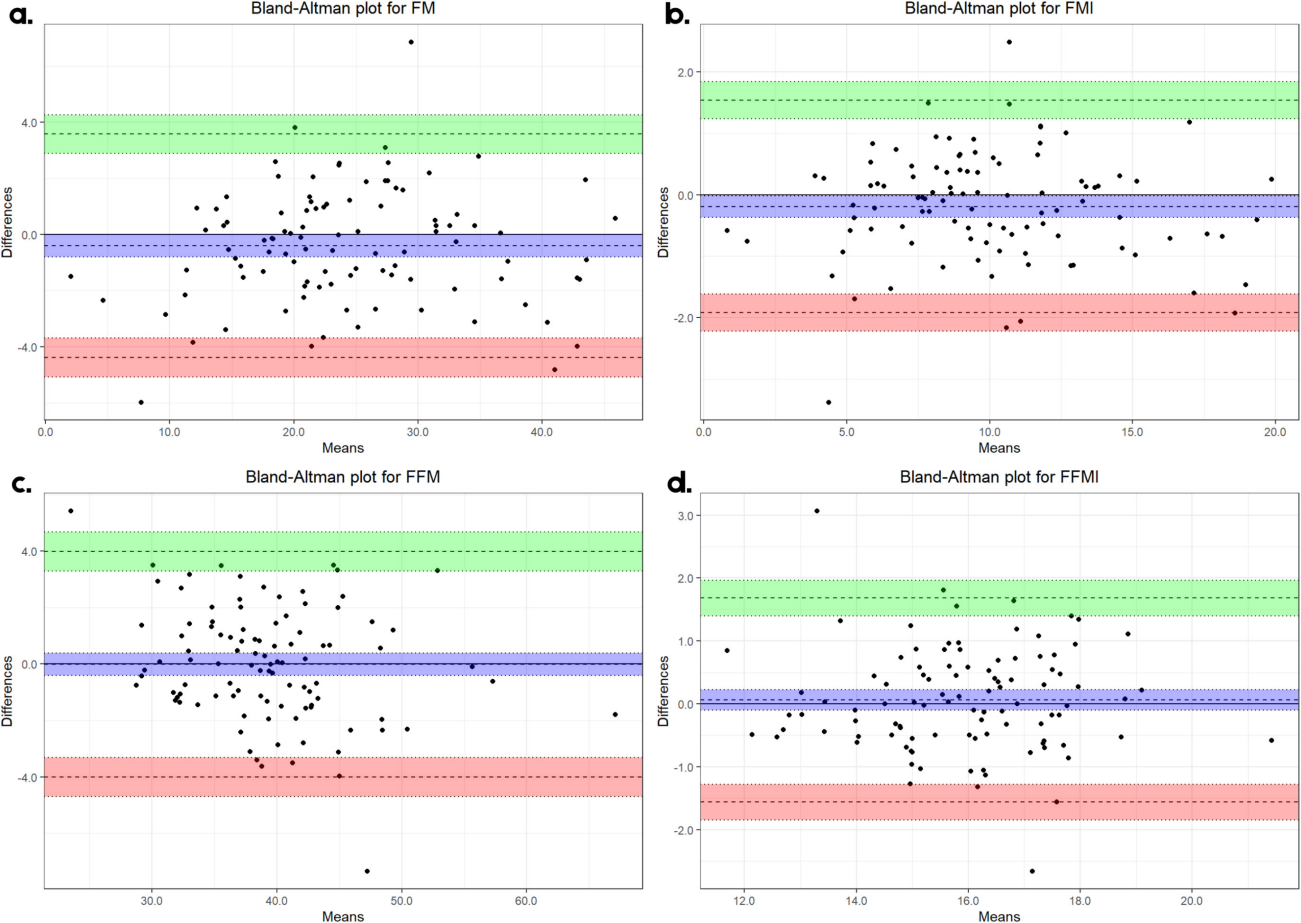
Bland–Altman plot of difference and means of body composition components measured by DXA and BIA: (a) total fat mass (FM), (b) fat mass index (FMI), (c) total fat‐free mass (FFM) and (d) fat‐free mass index (FFM).

In addition, Bland–Altman plots (Figures 2 and 3) illustrate the mean difference (±Δ) between DXA and BIA measurements as well as the mean of the two methods. The Δ values represent the Bland–Altman mean bias between methods, expressed in kilograms (kg) or kilograms per square metre (kg/m^2^), as appropriate. In the Bland–Altman plots, the central dashed line illustrates this mean systematic difference (Δ), whereas the outer dashed lines indicate the 95% limits of agreement and the shorter dashed lines represent the confidence interval surrounding the mean bias. All distributions showed homogeneity for the assessment of muscle mass (ASM, ASMI and ASM/BMI), and BIA underestimated values compared to DXA [Δ = 0.803 kg (95% CI: 0.518, 0.188), Δ = 0.363 kg/m^2^ (95% CI: 0.239, 0.487), Δ = 0.032 (95% CI: 0.019, 0.045), respectively]. In the case of fat mass, there was an overestimation of FM and FMI, but with differences very close to 0 in both cases. Notably, FFM and FFMI measurements showed the smallest differences between DXA and BIA, with the difference close to zero and its confidence interval indicating neither underestimation nor overestimation (Figure 3a,b).

### 3.3 Assessment of Muscle Mass and Myopenia

The mean values of muscle mass by ASM values by DXA were 23.6 ± 4.3 kg for males and 16.0 ± 2.4 kg for females, whereas the mean values of ASM by BIA were 22.7 ± 3.2 kg for males and 15.3 ± 2.7 kg for females. The mean values of ASMI by DXA were 7.9 ± 1.0 kg/m^2^ for men and 6.7 ± 0.8 kg/m^2^ for women, whereas the mean values of ASMI by BIA were 7.5 ± 0.6 kg/m^2^ for females and 6.3 ± 0.9 kg/m^2^ for males. Details of ASM/BMI values and prevalence of myopenia by gender are described in Table 3.

**TABLE 3:** Evaluation of muscle mass by gender.

	Male (*n* = 9)*		Female (*n* = 91)*	
	DXA	BIA	DXA	BIA
Appendicular skeletal muscle mass (ASM), kg	23.6 ± 4.3	22.7 ± 3.2	16.0 ± 2.4	15.3 ± 2.7
Myopenia by ASM	30%	45%	11%	22%
ASM index (ASMI), kg/m^2^	7.9 ± 1.0	7.5 ± 0.6	6.7 ± 0.8	6.3 ± 0.9
Myopenia by ASMI	9%	18%	11%	0%
ASM divided by BMI (ASM/BMI), kg/m^2^	1.0 (0.8–1.2)	1.0 (0.9–1.1)	0.6 (0.5–0.7)	0.6 (0.5–0.7)
Myopenia by ASM/BMI	11%	22%	11%	11%

*Note:* *Values in mean ± standard deviation; frequency (%) or median [interquartile range (25–75)].

Abbreviations: ASM, appendicular skeletal muscle mass; ASM/BMI, appendicular skeletal muscle mass divided by body mass index; ASMI, appendicular skeletal muscle mass index; BIA, bioelectrical impedance analysis; DXA, dual‐energy X‐ray absorptiometry; *n*, number.

When applying the sex‐specific thresholds established by the EWGSOP2 and FNIH, the BIA criteria for ASM, ASMI and ASM/BMI yielded sensitivities ranging from 89% to 100%. These findings indicate a robust capacity of BIA to identify positive myopenia cases relative to the DXA reference standard within the total sample (*n* = 100). On the other hand, the specificity for negative cases ranged from 67% for ASMI to 88% for ASM/BMI. Table 4 shows the sensitivity/specificity results for the detection of myopenia using the same parameter between BIA and DXA.

**TABLE 4:** Sensitivity and specificity for myopenia detection using BIA and DXA.

	Sensitivity	Specificity	PP	PN
Myopenia by BIA (ASM)	96%	78%	63%	98%
Myopenia by BIA (ASMI)	89%	67%	38%	96%
Myopenia by BIA (ASM/BMI)	100%	88%	52%	100%

*Note:* Values are expressed as frequency (%).

Abbreviations: ASM, appendicular skeletal muscle mass; ASM/BMI, appendicular skeletal muscle mass divided by body mass index; ASMI, appendicular skeletal muscle index; BIA, bioelectrical impedance analysis; DXA, dual‐energy X‐ray absorptiometry; NPV, negative predictive value; PPV, positive predictive value.

**FIGURE 4:**
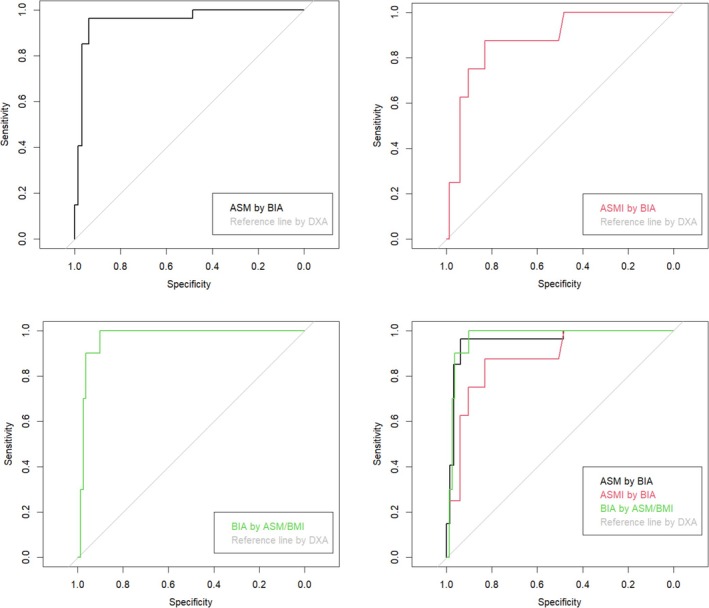
Receiver Operating Characteristic (ROC) curve analyses for the detection of myopenia in women with systemic sclerosis, comparing bioelectrical impedance analysis (BIA) and dual‐energy X‐ray absorptiometry (DXA) measurements of appendicular skeletal muscle (ASM, black) appendicular skeletal muscle index (ASMI, red) and appendicular skeletal muscle adjusted for body mass index (ASM/BMI, green). The AUCs were 0.956 (95% CI: 0.908–1.000) for ASM, 0.878 (95% CI: 0.763–0.993) for ASMI and 0.969 (95% CI: 0.935–1.000) for ASM/BMI. Optimal cut‐offs determined by the Youden index were 14.29 kg for ASM (SE = 96.3%, Sp = 93.8%, PPV = 86.7%, NPV = 98.4%), 5.78 kg/m^2^ for ASMI (Se = 87.5%, Sp = 83.1%. PPV = 33.3%, NPV = 98.6%) and 0.506 for ASM/BMI (Se = 100%, Sp = 90.1%, PPV = 55.6%, NPV = 100%). All analyses were performed in female participants due to the small number of men in the cohort.

Regarding the ability to assess myopenia, ROC curve analyses were performed to compare the discriminative ability of BIA and DXA measurements for ASM, ASMI and ASM/BMI, as shown in Figure 4. The AUC for ASM was 0.956 (95% CI: 0.908–1.000), for ASMI it was 0.878 (95% CI: 0.763–0.993) and for ASM/BMI it was 0.969 (95% CI: 0.935–1.000). According to the Youden index, the optimal cut‐offs were 14.29 kg for ASM (sensitivity 96.3%, specificity 93.8%, PPV 86.7%, NPV 98.4%), 5.78 kg/m^2^ for ASMI (sensitivity 87.5%, specificity 83.1%, PPV 33.3%, NPV 98.6%) and 0.506 for ASM/BMI (sensitivity 100%, specificity 90.1%, PPV 55.6%, NPV 100%). Finally, these analyses were restricted to female patients, and no men were included.

The corresponding 2 × 2 contingency tables for each muscle mass index (ASM, ASMI and ASM/BMI), based on guideline‐derived cut‐offs and including the Youden‐index–derived female‐specific cut‐off (0.506), are presented in Table S1. These analyses were performed in the total sample (*n* = 100) and were restricted to women for the Youden‐index threshold, as the limited number of male participants precluded sex‐specific validation.

Internal calibration analyses using 1000 bootstrap resamples showed adequate model calibration for all three indices (ASM, ASMI and ASM/BMI). The BCa 95% confidence intervals for calibration intercepts consistently included 0, and those for calibration slopes included 1, indicating no systematic over‐ or underestimation of risk. The median intercept and slope values for ASM were 0.049 (95% CI −0.90 to 1.32) and 1.06 (95% CI 0.54 to 2.21), respectively; for ASMI, 0.15 (95% CI −2.10 to 3.16) and 1.08 (95% CI 0.39 to 2.95); and for ASM/BMI, 0.09 (95% CI −1.82 to 4.72) and 1.06 (95% CI 0.41 to 4.12). These results suggest adequate internal calibration, despite wide confidence intervals reflecting sample‐size limitations.

## 4 Discussion

The main findings of this study are as follows: (1) BIA is a valid tool for assessing body composition in patients with SSc. The results demonstrated excellent concordance (CCC > 0.900) for muscle mass (ASM/BMI), FM and FMI and total FFM, and good agreement (CCC > 0.750) for muscle mass (ASM) and FFMI when compared with dual‐energy X‐ray absorptiometry. (2) In contrast, BIA showed a slight difference (very close to zero), with no tendency to overestimate or underestimate FFM and FFMI compared to DXA. (3) For the diagnosis of myopenia, ASM/BMI showed the best sensitivity/specificity ratio, supporting the use of BIA in clinical practice for the diagnosis of myopenia.

Several studies have compared body composition assessment by BIA and DXA across different scenarios and populations; however, limitations remain regarding device models, equipment brands and predictive equations, making it difficult to extrapolate findings to other populations [32, 33]. In patients with SSc, our results corroborate those of Spanjer et al. [14], who found good and excellent reliability using two different equations to assess BIA versus DXA. The authors evaluated FM and FFM in total kilograms (kg) and percentage (%). The results for percentage were good (ICC between 0.750 and 0.900). For quantity in kg, the ICCs were excellent, as the manufacturer's equation showed ICC = 0.908 (95% CI: 0.712, 0.959) and 0.956 (95% CI: 0.921, 0.974) for FM (kg) and FFM (kg), respectively. Using the Geneva equation [34], the reliability was ICC = 0.954 (95% CI: 0.910, 0.974) for FM (kg) and 0.971 (95% CI: 0.955, 0.982) for FFM (kg).

In contrast to the study by Spanjer et al. [14], our analysis distinguished FFM from skeletal muscle mass when interpreting body composition results. Because FFM includes both lean tissue and bone mineral content, it may confound estimates of muscle quantity and lead to misclassification of myopenia risk. This distinction is clinically relevant, as individuals with apparently preserved FFM may still exhibit reduced skeletal muscle mass and impaired muscular performance. Therefore, assessing ASM specifically is essential for an accurate diagnosis of myopenia, in accordance with current consensus recommendations. By including a comprehensive set of body composition parameters and proposing an SSc‐specific cut‐off, our study provides novel evidence supporting the clinical applicability of BIA in this context. In addition, the use of a different multifrequency BIA model in the present study contributes information on device‐specific performance and supports the generalizability of our findings. Overall, our results reinforce the relevance of appendicular skeletal muscle indices, a key component of sarcopenia that has been reported to affect approximately 22% (95% CI: 17%, 28%) of patients with SSc, based on systematic review data [12]. In that review, only two studies used BIA to assess muscle mass as a component of sarcopenia. Siegert et al. [35] reported a sarcopenia prevalence of 22%, whereas Sari et al. [36] identified 13.9% of patients with low muscle mass, which represents only one criterion of sarcopenia and may therefore underestimate its true prevalence in SSc.

An important historical limitation of BIA compared to DXA studies is Pearson's correlation coefficients and paired *t*‐tests. These analyses measure pairwise comparisons or are a measure of correlation and are not ideal measures of agreement and reliability. Additionally, the values of clinically relevant differences between BIA and DXA remain unclear, making it difficult to transpose them into clinical practice [37]. BIA usually tends to overestimate FFM or muscle mass and underestimate FM and FM percentage [38, 39]. This tendency may be explained by the influence of hydration status and dietary intake prior to BIA assessment, but a study by Mundstock et al. [40] showed that consuming a low number of calories from daily life food and beverages does not alter BIA body composition in healthy individuals.

BIA is an accessible method of assessing body composition using low‐frequency electrical current, whereas DXA is a method of assessing body composition using X‐ray energy. Although DXA is widely used in research settings, its clinical applicability is limited by restricted access and variability between device models [41]. For muscle mass, there are even more limitations because not all machines perform this segmental analysis and there are differences in nomenclature: total skeletal muscle mass (SMM) and ASM, making it difficult to compare with DXA [38]. As an alternative, newer BIA models provide segmental analysis of appendicular lean mass. Nevertheless, variability among the DXA models used for comparison raises concerns about validity, given the reported overestimation or underestimation in ASM assessment [38].

Cheng et al. [38] evaluated 1587 older adults, of which 522 were evaluated by BIA InBody 720 and DXA (Hologic), and showed an overestimation of ASM and ASMI compared to DXA (+8.25 ± 1.8 kg and 3.11 ± 0.45 kg/m^2^, respectively). However, their study used a different total muscle mass (SSM) value than we used in our study, which is the same recommended by the EWGSOP and the Asian Working Group for Sarcopenia (AWGS). As a correction, the study proposed an equation [ASM_predicted_ = −1.893 + 0.592 (SMM_BIA_) + 1.295 (gender) + 0.121 (BMI)], which reduced the difference by 0.04 ± 0.82 kg for ASM and 0.02 ± 0.31 kg/m^2^ for ASMI. This indicates that BIA is a valid tool for analysing muscle mass in older adults when using a correction equation and demonstrates high accuracy in detecting myopenia and diagnosing sarcopenia. In light of these findings, the results of the present study should also be interpreted in consideration of the specificity of the BIA device used. The multifrequency prediction equations employed by the analyser are proprietary and not publicly available and therefore may differ from those of other commercially available devices.

To the best of our knowledge, no study has evaluated the muscle mass and diagnostic accuracy of BIA compared to DXA for the assessment of myopenia in patients with SSc. Studies from Reiter et al. [42] and Nielsen et al. [43] corroborate our findings on the use of ASM in BIA to diagnose myopenia in older adults. The optimal ASM/BMI cut‐off identified in our study (0.506 for women) falls within the range reported in population‐based studies. Previous investigations in healthy or community‐dwelling women have proposed thresholds of 0.49/0.50 [29, 44, 45]. The proximity of our cut‐off to these values supports its physiological plausibility. The slightly lower threshold observed in our cohort may reflect disease‐specific alterations in body composition typical of SSc, such as oedema, tissue fibrosis and microvascular dysfunction, which can affect electrical conductivity and muscle density.

These findings reinforce our previous observations on the use of BIA, as studies have already used BIA to assess muscle mass and diagnose sarcopenia in older adults. Standardizing preassessment conditions, particularly hydration and nutritional status, is essential to minimize false‐positive or false‐negative classifications and improve reproducibility. Moreover, differences between device brands, models, predictive equations and measurement protocols should be considered when interpreting and comparing results across studies [42, 43]. Long‐term monitoring with periodic assessments is vital to confirm cases of myopenia. Patients may then be referred to robust methods like DXA and guided toward multiprofessional interventions, including nutritional support and exercise [46]. Future studies with periodic re‐evaluations and BIA cut‐offs in SSc are needed to validate data consistency and enhance clinical applicability.

As a limitation, we acknowledge that the inclusion of patients by invitation may introduce some bias into our sample (long duration of disease and low Rodnan score), with a low prevalence of myopenia. Moreover, our predominantly white, single‐centre sample limits generalizability to other ethnic and geographic populations. The limited number of men in our study (*n* = 9) did not allow for the establishment of sex‐specific cut‐offs or accurate estimation of diagnostic accuracy parameters, reflecting the epidemiological profile of SSc, which predominantly affects women. However, it is believed that this did not affect our main results, which were designed as a validation study. Although internal bootstrap validation (1000 resamples) demonstrated adequate calibration across all models, the wide confidence intervals indicate some degree of uncertainty, likely related to sample size and event frequency. As the primary objective of this study was not to develop or validate predictive models, but rather to compare diagnostic accuracy and determine optimal cut‐off points, these results should be interpreted as exploratory. Future studies should evaluate the reproducibility of our findings using different BIA analysers and models, validate sex‐specific BIA cut‐offs and explore equation‐independent approaches such as raw impedance vectors or phase angle to improve external validity and comparability across devices.

In the clinical context, although SSc can affect body fluid distribution through oedema and microvascular alterations, all participants were clinically stable, and no cases of significant oedema were observed during the assessments, minimizing potential bias in impedance measurements. In the case of metal joint prostheses, it is recognized that there is a lack of consensus in the literature regarding BIA‐derived assessments of body composition due to the greater conductivity of metal, which may influence the results (Table S2). Nevertheless, a sensitivity analysis excluding patients with metal prostheses yielded consistent CCC classifications. Likewise, although our sample predominantly consisted of patients with controlled disease, mainly those with the limited cutaneous subtype, our stratified analyses demonstrated that the CCC remained consistent across different disease subtypes, supporting the robustness of our findings.

In conclusion, this study demonstrates the feasibility of electrical bioimpedance analysis as a valid and practical tool for assessing and monitoring fat mass, FFM and muscle mass in patients with systemic sclerosis. Assessment of ASM adjusted for BMI (ASM/BMI) by BIA demonstrated the highest concordance and diagnostic accuracy compared to DXA, with a proposed cut‐off point of 0.506 for women. The effectiveness and low cost of diagnosing myopenia are crucial for multidisciplinary management, including the identification of optimal strategies for planning physical activity, physical exercise and nutrition. This may facilitate significant benefit for patients, including the preservation of a functional musculoskeletal system and functional capacity, which are key targets for damage in SSc.

## Funding

We thank the Hospital de Clínicas de Porto Alegre (HCPA; https://ror.org/010we4y38) for the financial support received from the Financiamento e Incentivo à Pesquisa (FIPE). This study was also supported by the Coordenação de Aperfeiçoamento de Pessoal de Nível Superior — Brazil (CAPES) – Finance Code 001 and by the Rheumatology Society of Rio Grande do Sul (SRRS) – Research Support Fund (FAPE).

## Ethics Statement

This study received Institutional Review Board (IRB) approval and is registered under number 2021‐0504.

## Conflicts of Interest

The authors declare no conflicts of interest.

## Supporting information


**Table S1:** 2 × 2 diagnostic classification tables comparing bioelectrical impedance analysis (BIA) and dual‐energy X‐ray absorptiometry (DXA) for the detection of myopenia in patients with systemic sclerosis.

**Table S2:** Comparison between DXA and BIA measurements.

## Data Availability

De‐identified paired BIA–DXA data and SPSS syntax used for statistical analyses are available in a public repository (Dryad, DOI to be provided upon acceptance). These materials include all variables used for the agreement and diagnostic accuracy analyses, allowing external validation and replication in accordance with STARD and FAIR data‐sharing principles.
