# Supplementary material for: Validity of Bioelectrical Impedance Analysis for the Assessment of Body Composition in Patients With Systemic Sclerosis
Source: J Cachexia Sarcopenia Muscle. 2026 Mar 26;17(2):e70273. doi: 10.1002/jcsm.70273 (PMC13054670; doi:10.1002/jcsm.70273)
Supplement: Supplementary file 1 — Table S1: 2 × 2 diagnostic classification tables comparing bioelectrical impedance analysis (BIA) and dual‐energy X‐ray absorptiometry (DXA) for the detection of myopenia in patients with systemic sclerosis. [file JCSM-17-e70273-s001.docx]

**Supplementary Table 1. 2×2 diagnostic classification tables comparing bioelectrical impedance analysis (BIA) and dual-energy X-ray absorptiometry (DXA) for the detection of myopenia in patients with systemic sclerosis.**

|  | **DXA**  **Myopenia (+)** | | **DXA**  **No Myopenia (−)** | | **Total** | **Sensitivity** | **Specificity** | **PPV** | **NPV** |
| --- | --- | --- | --- | --- | --- | --- | --- | --- | --- |
| **ASM (n = 100)**  BIA Myopenia (+)  BIA Myopenia (−) | | 27  1 | | 16  56 | 43  57 | 0.96 | 0.78 | 0.63 | 0.98 |
| **ASMI** **(n = 100)**  BIA Myopenia (+)  BIA Myopenia (−) | | 6  3 | | 10  81 | 16  84 | 0.67 | 0.89 | 0.38 | 0.96 |
| **ASM/BMI (n = 100)**  BIA Myopenia (+)  BIA Myopenia (−) | | 11  0 | | 10  79 | 21  79 | 1.00 | 0.89 | 0.52 | 1.00 |
| **ASM/BMI [cut-off = 0.506; women only (n = 91)]**  BIA Myopenia (+)  BIA Myopenia (−) | | 10  0 | | 8  73 | 18  73 | 1.00 | 0.90 | 0.56 | 1.00 |

Each 2×2 table presents the agreement between BIA and DXA for the diagnosis of myopenia based on appendicular skeletal muscle (ASM), appendicular skeletal muscle index (ASMI), and appendicular skeletal muscle adjusted for body mass index (ASM/BMI). The ASM, ASMI, and guideline-based ASM/BMI cut-offs were defined according to the EWGSOP2 and FNIH recommendations. The second ASM/BMI analysis (cut-off = 0.506; women only, n = 91) represents the optimal threshold derived in the present study using the Youden index. DXA served as the reference standard. BIA = bioelectrical impedance analysis (InBody 370S). DXA = dual-energy X-ray absorptiometry. Values represent observed frequencies and corresponding diagnostic performance metrics.
