# Supplementary material for: Validity of Bioelectrical Impedance Analysis for the Assessment of Body Composition in Patients With Systemic Sclerosis
Source: J Cachexia Sarcopenia Muscle. 2026 Mar 26;17(2):e70273. doi: 10.1002/jcsm.70273 (PMC13054670; doi:10.1002/jcsm.70273)
Supplement: Supplementary file 2 — Table S2: Comparison between DXA and BIA measurements. [file JCSM-17-e70273-s002.docx]

| **Supplementary Appendix 2. Comparison between DXA and BIA measurements** | | | | | | |
| --- | --- | --- | --- | --- | --- | --- |
|  |  | DXA* | BIA* | Δ (95% CI of Δ) | CCC (95% CI of CCC) | *p* |
| **Diffuse cutaneous** | ASM | 16.923 ± 2.890 | 16.143 ± 3.461 | 0.780 (0.030, 1.530) | 0.825⁺ (0.653, 0.917) | <0.001 |
|  | ASMI | 6.827 ± 0.889 | 6.445 ± 1.005 | 0.382 (0.215, 0.456) | 0.557 (0.223, 0.770) | <0.001 |
|  | ASM/BMI | 0.637 (0.592 – 0.754) | 0.603 (0.564 – 0.702) | 0.033 (-0.003, 0.070) | 0.754⁺ (0.529, 0.880) | <0.001 |
|  | FM | 23.142 ± 8.267 | 23.800 ± 7.398 | -0.658 (-1.504, 0.188) | 0.965⁺⁺ (0.926, 0.984) | <0.001 |
|  | FMI | 9.295 ± 3.250 | 9.600 ± 2.919 | -0.305 (-0.706, 0.096) | 0.950⁺⁺ (0.892, 0.977) | <0.001 |
|  | FFM | 39.534 ± 5.803 | 39.309 ± 6.312 | 0.225 (-0.657, 1.107) | 0.943⁺⁺ (0.874, 0.975) | <0.001 |
|  | FFMI | 15.961 ± 1.661 | 15.792 ± 1.664 | 0.169 (-0.230, 0.569) | 0.841⁺ (0.663, 0.929) | <0.001 |
| **Limited cutaneous** | ASM | 16.726 ± 3.435 | 15.992 ± 3.339 | 0.734 (0.465, 1.004) | 0.930⁺⁺ (0.888, 0.957) | <0.001 |
|  | ASMI | 6.707 ± 0.934 | 6.389 ± 0.870 | 0.318 (0.210, 0.422) | 0.845⁺ (0.764, 0.900) | <0.001 |
|  | ASM/BMI | 0.636 (0.540 – 0.732) | 0.602 (0.537 – 0.704) | 0.028 (0.016, 0.039) | 0.953⁺⁺ (0.924, 0.971) | <0.001 |
|  | FM | 24.751 ± 9.254 | 25.062 ± 9.552 | 0.312 (−0.787, 0.164) | 0.980⁺⁺ (0.96, 0.987) | <0.001 |
|  | FMI | 10.120 ± 4.026 | 10.254 ± 4.206 | 0.134 (-0.336, 0.068) | 0.981⁺⁺ (0.969, 0.989) | <0.001 |
|  | FFM | 39.318 ± 6.584 | 39.428 ± 6.778 | 0.110 (-0.578, 0.359) | 0.962⁺⁺ (0.938, 0.978) | <0.001 |
|  | FFMI | 15.804 ± 1.776 | 15.804 ± 1.750 | 0.000 (-0.182, 0.181) | 0.919⁺⁺ (0.869, 0.951) | <0.001 |
| **Sine scleroderma** | ASM | 16.621 ± 3.869 | 15.522 ± 4.157 | 1.099 (-0.046, 2.245) | 0.824⁺ (0.584, 0.931) | <0.001 |
|  | ASMI | 6.916 ± 0.838 | 6.409 ± 1.019 | 0.506 (0.020, 0.992) | 0.452 (0.031, 0.733) | <0.001 |
|  | ASM/BMI | 0.662 (0.572 – 0.745) | 0.605 (0.529 – 0.720) | 0.048 (-0.003, 0.099) | 0.855⁺ (0.658, 0.942) | <0.001 |
|  | FM | 23.541 ± 10.171 | 23.913 ± 9.408 | 0.371 (-1.852, 1.109) | 0.959⁺⁺ (0.892, 0.984) | <0.001 |
|  | FMI | 9.939 ± 4.271 | 10.180 ± 4.173 | 0.241 (-0.865, 0.383) | 0.959⁺⁺ (0.903, 0.986) | <0.001 |
|  | FFM | 39.243 ± 6.961 | 39.213 ± 8.375 | 0.031 (-1.464, 1.525) | 0.939⁺⁺ (0.845, 0.972) | <0.001 |
|  | FFMI | 16.414 ± 1.562 | 16.284 ± 1.762 | 0.131 (-0.460, 0.721) | 0.776⁺ (0.479, 0.914) | <0.001 |
| **Without patients with metallic prostheses** | FFMI | 16.744 ± 3.382 | 15.879 ± 3.461 | 0.865 (0.580, 1.150) | 0.887⁺ (0.840, 0.923) | <0.001 |
|  | ASMI | 6.775 ± 0.911 | 6.389 ± 0.932 | 0.386 (0.261, 0.512) | 0.712 (0.603, 0.793) | <0.001 |
|  | ASM/BMI | 0.623 (0.553 – 0.709) | 0.586 (0.527 – 0.687) | 0.035 (0.022, 0.048) | 0.902⁺⁺ (0.859, 0.933) | <0.001 |
|  | FM | 24.171 ± 9.209 | 24.601 ± 9.118 | -0.430 (-0.844, -0.017) | 0.974⁺⁺ (0.962, 0.982) | <0.001 |
|  | FMI | 9.907 ± 3.909 | 10.115 ± 3.952 | -0.208 (-0.387, -0.029) | 0.973⁺⁺ (0.963, 0.982) | <0.001 |
|  | FFM | 39.333 ± 6.518 | 39.296 ± 7.006 | 0.037 (-0.377, 0.451) | 0.959⁺⁺ (0.933, 0.969) | <0.001 |
|  | FFMI | 15.955 ± 1.745 | 15.881 ± 1.756 | 0.074 (-0.095, 0.243) | 0.886⁺ (0.834, 0.922) | <0.001 |
| *CCC: concordance correlation coefficient; p: p-value of CCC.*  *⁺ CCC = 0.750–0.900 (good concordance); ⁺⁺ CCC > 0.900 (excellent concordance).*  **Values are presented as mean ± standard deviation or median (interquartile range, 25–75) as appropriate.*  *Δ: mean difference between DXA and BIA measurements.* | | | | | | |
